# Integrated metabolomic and transcriptomic analyses reveal distinct flavonoid biosynthetic pathways underlying petal color diversity in *Meconopsis*

**DOI:** 10.3389/fpls.2026.1743820

**Published:** 2026-02-17

**Authors:** Sitong Qiao, Anqi Ding, Jiyang Wang, Mengting Li, Leixin Deng, Hongqiang Lin, Hangcheng Hu, Meng Tang, Shujie Tang, Duwei Xia, Haoran Jin, Guoyan Wang

**Affiliations:** 1 College of Geography and Planning, Chengdu University of Technology, Chengdu, Sichuan, China; 2 Research Center of National Park, Sichuan Key Research Base for Social Sciences, Chengdu, Sichuan, China; 3 Human Geography Research Center of Qinghai-Tibet Plateau and Its Eastern Margin, Chengdu, Sichuan, China; 4 Sichuan Wolong National Natural, Reserve Administration Bureau, Wenchuan, Sichuan, China

**Keywords:** anthocyanin, flavonol, flower coloration, *Meconopsis*, metabolomics, transcriptomics

## Abstract

**Introduction:**

Flower color is a key ornamental and ecological trait that influences both aesthetic appeal and pollinator interactions. Although the biosynthetic and regulatory mechanisms of floral pigmentation are well characterized in several model species, they remain poorly understood in *Meconopsis*, an alpine genus renowned for its striking color diversity. Elucidating the molecular basis of petal coloration is crucial for the genetic improvement and conservation of this unique ornamental resource.

**Methods:**

Here, we combined metabolomic and transcriptomic analyses to investigate the molecular mechanisms underlying flower coloration in three *Meconopsis* species—*M. balangensis* (blue), *M. punicea* (red), and *M. integrifolia* (yellow)—using *M. argemonantha* (white) as a control.

**Results:**

Metabolite profiling revealed strong correlations between color parameters and pigment composition, particularly flavonoids and anthocyanins. Blue and red pigmentation were primarily attributed to cyanidin- and delphinidin-based anthocyanins, while yellow coloration resulted from quercetin derivatives. Transcriptome analysis identified key structural genes (*F3’H*, *DFR*, *ANS*, *UFGT*, *CHS*, *F3H*, and *FLS*) and regulatory transcription factors (MYB and bHLH) that collectively modulate flavonoid biosynthesis across species.

**Discussion:**

Our findings demonstrate that divergence in the regulation of the flavonoid biosynthetic pathway drives color differentiation among *Meconopsis* species. This study provides new insight into the metabolic and transcriptional control of alpine flower coloration and establish a theoretical foundation for the molecular breeding of novel *Meconopsis* cultivars.

## Introduction

Flower color is one of the most visually striking traits in plants and plays crucial ecological and evolutionary roles by attracting pollinators and influencing reproductive success (Yin et al., 2021; Sagheer et al., 2022). The molecular mechanisms governing floral pigmentation have been extensively studied in several model and ornamental plants; however, they remain poorly understood in *Meconopsis*, an alpine genus celebrated for its remarkable diversity of flower colors. *Meconopsis* is a rare and ecologically important genus distributed primarily in the high-altitude regions of China (2,500stitud m) (Xiao and Simpson, 2017). Owing to its vibrant yellow, red, purple, blue, and occasionally white flowers, *Meconopsis* holds great ornamental value and is also recognized for its medicinal and ecological significance (Chen et al., 2023). The vibrant petal coloration and dense pubescence on the leaves and stems of *Meconopsis* are critical adaptations to high-altitude environments, contributing to UV radiation resistance, mechanical protection, and thermal insulation (Qu et al., 2019). Moreover, a long-term adaptive relationship has evolved between floral color and pollinator behavior. Different pollinator groups display distinct color preferences. For example, bees predominantly forage on blue to purple flowers, while flies exhibit a marked preference for yellow-flowered plants (Nie et al., 2025). These adaptive traits confer *Meconopsis* with significant potential for horticultural improvement and landscape use (Yu et al., 2020).

The development of floral coloration is regulated by multiple factors, including petal epidermal morphology, anthocyanins, flavonoids, pH levels, metal ions, and environmental conditions (Zhao and Tao, 2015). Flavonoids, carotenoids, and alkaloids are the primary metabolites contributing to petal coloration (Grotewold, 2006). Among these, flavonoid pigments are the most extensively studied secondary metabolites. Flavonoids mainly comprise flavones, flavonols, and anthocyanin glycosides, which together produce a wide range of petal colors (Winkel Shirley, 2001; Iwashina, 2015). Anthocyanins generally produce red to blue pigmentation, whereas flavonols and flavones contribute to white and yellow hues in petals (Masahiro et al., 2024). In *Pericallis hybrida*, blue petals primarily accumulate delphinidin with trace amounts of cyanidin (Jin et al., 2016). The yellow pigmentation of *Camellia nitidissima* mainly results from quercetin-3-O-β-D-glucoside and quercetin-7-O-β-D-glucoside (Zhou et al., 2013).

The accumulation of anthocyanins and flavonols affects both flower development and color variation (Weiss, 2000; Wang et al., 2021). Anthocyanin and flavonol biosynthesis together form the core pathway of flavonoid metabolism (Tan et al., 2018). The flavonoid biosynthetic pathway has been well characterized in *Arabidopsis thaliana*, *Petunia hybrida*, and *Antirrhinum majus* (Martin et al., 1991; Koes et al., 2005; Grotewold, 2006; Nakatsuka et al., 2014). Flavonoid metabolism begins with enzymatic reactions from phenylalanine, ultimately producing flavones, flavonols, and anthocyanins (Liu et al., 2021). Early flavonoid biosynthesis involves genes such as *chalcone synthase (CHS)*, *chalcone isomerase (CHI)*, *flavanone 3-hydroxylase* (*F3H*), *flavonoid 3’-hydroxylase (F3’H)*, and *flavonoid 3′,5′-hydroxylase (F3′5′H)*. Late anthocyanin biosynthesis is governed by *dihydroflavonol reductase* (*DFR*), *anthocyanidin synthase* (*ANS*), and *UDP-glucose: flavonoid 3-O-glucosyltransferase* (*UFGT*) (Fraser and Chapple, 2011; Peng et al., 2021).

Flavonoid biosynthesis is transcriptionally regulated by transcription factors (TFs) that bind to specific cis-acting elements within target gene promoters. The regulatory mechanism of flavonoid biosynthesis has been well established, with the conserved MBW complex—comprising MYB, basic helix–loop–helix (bHLH), and WD40-repeat proteins—playing a central role (Antonio et al., 2008). Other transcription factor families, including bZIP, WRKY, and ERF, have also been implicated in regulating flavonoid biosynthesis (An et al., 2018; Gu et al., 2024). Among these, MYB and bHLH are the most extensively studied (Liu et al., 2025).

Several studies have examined the floral coloration and environmental adaptability of *Meconopsis* (Qu et al., 2022). Flavonoids are the primary pigments in *Meconopsis*, and key biosynthetic genes such as *UFGT* and *FLS* have been identified (Tanaka et al., 2001; Qu et al., 2019; Wang et al., 2024). Additionally, floral color formation in *Meconopsis* has been shown to depend on multiple factors, including petal pH, metal ions (Mg²^+^ and Fe²^+^), and UV radiation (Yoshida et al., 2006; Ou et al., 2024). However, *Meconopsis* includes diverse species with a broad spectrum of flower colors. The developmental, regulatory, and biochemical mechanisms underlying petal color variation remain poorly understood. Therefore, *M. balangensis*, *M. punicea*, and *M. integrifolia*, bearing blue, red, and yellow petals respectively, were selected to investigate the molecular mechanisms of petal coloration. Petals at three developmental stages were analyzed for epidermal cell morphology, phenotypic traits, pigment composition, pH, and major pigments contributing to coloration, to elucidate potential transcriptional regulatory mechanisms. This study identifies candidate genes and provides a theoretical foundation for the molecular breeding of *Meconopsis*.

## Materials and methods

### Plant materials

The sampling of *Meconopsis* petals was approved by the Administration Bureau of Sichuan Wolong National Nature Reserve. Petals of *M. balangensis*, *M. punicea*, and *M. integrifolia* were used as experimental materials. Samples were selected at five flowering stages (S1–S5) (**Figure 1A**) in June 2023 from approximately 4,000 m elevation in the Balang Mountain, Xiaojin County, Sichuan Province, China (102°53′E, 30°55′N). The five developmental stages included the early bud (S1), late bud (S2), early flowering (S3), full bloom (S4), and late flowering (S5) stages. A portion of the fresh petals was used to measure color parameters (L*, a*, b*, C*) and for scanning electron microscopy (SEM) observation. Remaining samples were immediately frozen in liquid nitrogen and stored at −80 °C.

**Figure 1:**
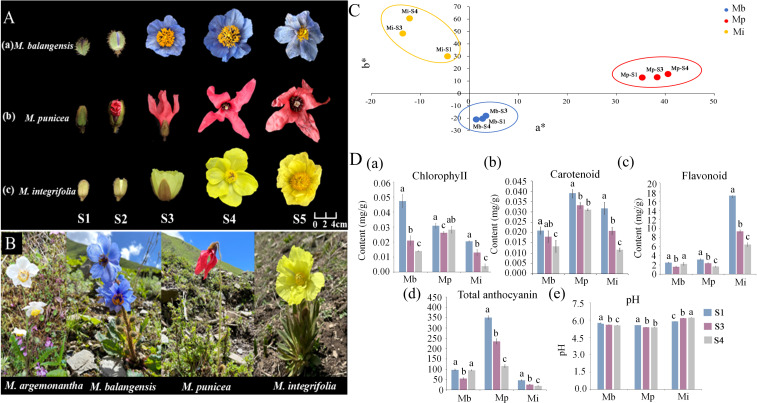
Petal developmental stages, distribution of hue values and five physiological indice values of different *Meconopsis* species. **(A)** Five stages petals of three *Meconopsis* species. **(B)** Petals in full bloom of four *Meconopsis* species. **(C)** Distribution of hue a* and b* values among three flower colors of *Meconopsis*. **(D)** Five physiological indices of petals for three *Meconopsis* species across three developmental stages: (a) Chlorophyll content (mg/g); (b) Carotenoid content (mg/g); (c) Flavonoid content (mg/g); (d) Total anthocyanin content (μg/g); (e) pH value. Different letters (a, b, c) indicate significant differences among the three developmental stages (*p* < *0.05*).

Petals of *M. argemonantha* at full bloom (**Figure 1B**) were collected from approximately 4,200 m elevation on Zhari Mountain, Longzi County, Shannan City, Tibet Autonomous Region, China (92°58′E, 28°40′N) Samples were immediately frozen in liquid nitrogen after collection, transported to the laboratory, and stored at −80 °C. The four species were abbreviated as Mb, Mp, Mi, and Ma, respectively.

### Measurement of phenotypic parameters

Color phenotypes of *Meconopsis* tepals at three developmental stages were measured using a colorimeter (CM-2600d, Konica Minolta, Japan). Lightness (L*) and hue values (a* and b*) were recorded for each petal sample. L*, a*, and b* represent the three coordinates in the CIE Lab color space, collectively describing color appearance. L* represents brightness ranging from black (0) to white (100); a* ranges from red (positive) to green (negative); b* ranges from yellow (positive) to blue (negative). Chroma (C*) was calculated using the formula C* = (a² *+ b*²)¹^/^², representing color saturation (Gonnet, 2001). Five petals from each plant were measured three times. The mean of five plants was used as the representative color value for each species.

### Scanning electron microscopy observation of petals

Petals at full bloom were cut into 5 mm × 5 mm squares and immediately immersed in 3% glutaraldehyde fixative. Fixed samples were washed three times with ultrapure water (UP) for 10 min each. After post-fixation with 1% osmium tetroxide for 1 h, the samples were again washed three times with UP water for 10 min each. Samples were dehydrated through a graded ethanol series (30%, 50%, 70%, 80%, 90%, 95%, 100%), with each step lasting 15 min. Samples were dried using a critical-point dryer, mounted on stubs with conductive adhesive and sputter-coated using an ion coater. Finally, the samples were examined and imaged using a scanning electron microscope (SEM) (He et al., 2021).

### Quantification of chlorophyll, carotenoid, flavonoid, and total anthocyanin contents

Chlorophyll and carotenoid contents in *Meconopsis* tepals were quantified using a plant chlorophyll content detection kit (Jiancheng, Nanjing, China) according to the manufacturer’s protocol. Fresh petals were ground into fine powder under liquid nitrogen, and 0.05 g of the powder was used. Anhydrous ethanol and acetone were mixed at a 1:2 (v/v) ratio to prepare the extraction solution. To the prepared sample, 0.5 mL distilled water and 50 mg of Reagent 1 were added and mixed thoroughly. The total volume was adjusted to 2.5 mL, and samples were extracted in the dark for approximately 3 h until the residue turned white, indicating complete extraction. The mixture was centrifuged at 4,000 rpm for 10 min. Using the extraction solution as a blank, absorbance values were measured at 470, 663, and 645 nm, denoted as A_470_, A_663_, and A_645_, respectively. Three biological replicates were conducted for each sample. Chlorophyll and carotenoid contents were calculated as follows:


$$\text{Ca}\;\left(\text{mg}/\text{g}\right)=\left(12.7\times {\text{A}}_{663}-2.46\times {\text{A}}_{645}\right)\times \text{V}\times \text{F}÷\text{W}÷1000;$$

$${\text{C}}_{\text{b}}\left(\text{mg}/\text{g}\right)=\left(22.9\times {\text{A}}_{645}-4.68\times {\text{A}}_{663}\right)\times \text{V}\times \text{F}÷\text{W}÷1000;$$

$${\text{C}}_{\text{T}}\left(\text{mg}/\text{g}\right)=\left(20.21\times {\text{A}}_{645}+8.02\times {\text{A}}_{663}\right)\times \text{V}\times \text{F}÷\text{W}÷1000;$$

$$\text{Car }\left(\text{mg}/\text{g}\right)=\left[\left(1000\times {\text{A}}_{470}-3.27\times \text{Ca}-104\times {\text{C}}_{\text{b}}\right)÷229\right]\times \text{V}\times \text{F}÷\text{W}÷1000$$

Where Ca refers to chlorophyll a content, C_b_ refers to chlorophyll b content, C_T_ refers to total chlorophyll content, and Car refers to carotenoid content. V is the total volume of the extract (2.5 mL), F is the dilution factor (1), and W is the weight of the sample (0.05 g).

Flavonoid content was measured using a colorimetric flavonoid assay kit (Jiancheng, Nanjing, China) according to the manufacturer’s protocol. Petal samples were ground in liquid nitrogen, and ~0.05 g of powder was placed into a centrifuge tube. Subsequently, 2 mL of 60% ethanol was added, and samples were extracted with shaking at 60 °C for 2 h. After extraction, samples were centrifuged at 10,000 rpm for 10 min at 25 °C, and the supernatant was collected as the flavonoid extract. Subsequent procedures followed the manufacturer’s instructions. Three biological replicates were set up for each sample.

The total anthocyanin content in petals was determined using the Enzyme-linked Biological Total Anthocyanin Extraction Kit (Meilian, Shanghai, China), following the manufacturer’s instructions. A 0.1 g petal sample was mixed with 1 mL extraction solution, homogenized thoroughly, and transferred to an EP tube. The volume of the extraction solution was adjusted to 1 mL. The tube was tightly capped and subjected to extraction at 60 °C for 30 min with several shaking intervals. The sample was centrifuged at 12,000 rpm for 10 min at 25 °C, and the supernatant was collected. Subsequent operations were performed according to the kit instructions. Three biological replicates were set up for each sample.

### Measurement of petal pH

Petals of three color variants at three developmental stages (S1, S3, S4; **Figure 1A**) were frozen in liquid nitrogen and ground into powder. Then, 0.02 g of powdered petals was placed in a 1.5 mL tube, homogenized with 0.1 mL distilled water, and centrifuged at 12,000 rpm for 15 min at 4 °C. The pH of the supernatant was measured three times at 25 °C using a pH meter (Yuan et al., 2024). Five biological replicates were analyzed per species, each with four technical replicates.

### UPLC–MS/MS analysis

The petals at the full-bloom stage of the four flower colors were used as the experimental materials (**Figure 1B**), and they were crushed with a mixing grinder equipped with zirconia beads. A 0.1 g aliquot of the powdered sample was extracted. After centrifugation at 12,000 rpm for 10 min, the extract was filtered and analyzed using ultra-high-performance liquid chromatography (UPLC). All chromatographic separations were performed using an UltiMate 3000 UPLC system (Thermo Fisher Scientific, Bremen, Germany). A high-resolution Q Exactive™ hybrid quadrupole-Orbitrap mass spectrometer (Thermo Fisher Scientific) was used to detect metabolites eluted from the column. Quality control (QC) samples were prepared by mixing equal volumes of petal extracts from white, blue, red, and yellow *Meconopsis* morphotypes. Four biological replicates were included in this assay. Variable importance in projection (VIP) values, P-values, and fold changes (FC) were used to identify differentially accumulated metabolites (DAMs). Metabolites were considered significant with VIP ≥ 1, P< 0.05, and FC ≥ 2 or ≤ 0.5.

### RNA sequencing and data analysis

The same batch of full-bloom petals from four color varieties used for UPLC–MS/MS detection was submitted to Hangzhou Lianchuan Biotechnology (China) for RNA sequencing, with three biological replicates. Total RNA was extracted from petal samples using the RNAprep Pure Plant Kit (DP441, Tiangen, China) according to the manufacturer’s protocol and assessed for integrity, purity and concentration. cDNA libraries were sequenced on the Illumina NovaSeq™ 6000 platform to obtain raw reads. Quality control was applied to remove adapters, primers, and low-quality reads, generating high-quality clean data. Clean reads were *de novo* assembled using Trinity (v2.15) software to construct the unigene library. Transcript abundance was quantified using Salmon (v1.9.0) by calculating TPM (Transcripts Per Kilobase Million). Differential gene expression analysis was performed using edgeR (v3.40.2) between experimental groups and among individual samples. Functional annotation was conducted against the NR, Swiss-Prot, GO, COG, KOG, eggNOG, and KEGG databases. Differentially Expressed Genes (DEGs) were identified with thresholds set at FDR (False Discovery Rate)< 0.05 and log_2_|FC| ≥ 2.

### Quantitative real-time PCR analysis

Twelve genes were selected from each comparison for validation by quantitative real-time PCR (qRT-PCR) based on transcriptome data. Expression patterns of petals from three *Meconopsis* species at five stages (S1–S5; **Figure 1A**) were analyzed to investigate gene expression dynamics. Total RNA extraction followed the procedure described above. First-strand cDNA was synthesized from total RNA according to the instructions of FastKing RT Kit with gDNA (KR116, Tiangen, China). qPCR was performed with the following reaction parameters: 94°C for 20 s, 40 cycles of 94°C for 10 s and 60°C for 20 s, and 60C for 30 s. A 10 μL reaction system was used as follows: 1 μL cDNA template, 5 μL qPCR Master Mix (Lanyun, China), 0.2 μL forward primer, 0.2μL reverse primer, and 3.6 μL nuclease-free water. *GAPDH* was used as the reference gene for normalization (Wang et al., 2023). Primers were designed using Primer Premier 6 software. 2^-ΔΔCT^ method was used to normalize the relative expression of the DEGs (**Supplementary Table S1**). All qRT-PCR experiments were performed in triplicate. The plant material used for qRT-PCR validation was identical to that used in the transcriptomic analysis.

## Results

### Cell morphology and physiological parameter analysis

The petal epidermal cells of the three *Meconopsis* species at the full-bloom stage (S4; **Figure 1A**) exhibited similar overall morphology, with intact and elongated cell shapes (**Supplementary Figure S1**). Therefore, cell morphology in *Meconopsis* petals appeared to have no significant effect on flower coloration. The petal hues of the three *Meconopsis* species varied across three developmental stages (**Table 1**). In *M. balangensis* (Mb), L* values were 47.50, 50.57, and 48.16, indicating a change in blue intensity from deep to light and then returning to deep across the three developmental stages. The a* value of *M. punicea* (Mp) ranged from 35.29 to 40.55, and the L* value ranged from 34.21 to 44.58. In *M. integrifolia* (Mi), L* values decreased progressively from 86.49 to 79.19, while b* values increased steadily from 29.99 to 60.59 (**Figure 1C**).

**Table 1:** Phenotypic parameters values of *Meconopsis* petals.

Species	Stages	L^*^	a^*^	b^*^	C^*^
Mb	S1	47.50 ± 1.26b	2.71 ± 0.53a	-20.43 ± 0.89b	20.61 ± 0.95a
	S3	50.57 ± 1.07a	3.38 ± 0.29a	-18.07 ± 1.20a	18.38 ± 1.15b
	S4	48.16 ± 1.39ab	1.40 ± 0.27b	-21.00 ± 0.87b	21.05 ± 0.88a
Mp	S1	34.21 ± 0.79c	40.55 ± 0.90a	15.65 ± 0.34a	43.47 ± 0.80a
	S3	42.86 ± 0.34b	38.35 ± 1.01b	13.06 ± 0.83b	40.51 ± 1.22b
	S4	44.58 ± 1.15a	35.29 ± 0.62c	12.84 ± 0.46b	37.56 ± 0.70c
Mi	S1	86.49 ± 0.39a	-4.45 ± 0.17a	29.99 ± 0.69c	30.32 ± 0.68c
	S3	83.18 ± 0.31b	-13.59 ± 0.65c	48.38 ± 0.33b	50.25 ± 0.15b
	S4	79.19 ± 0.43c	-12.21 ± 0.38b	60.59 ± 0.90a	61.81 ± 0.94a

The asterisk (*) in L*, a*, and b* is part of the name. Different letters (a, b, c) indicate significant differences at different sampling stages (*p* < 0.05).

Pigment contents and pH values in the petals of the three *Meconopsis* species are shown in **Figure 1D**. In Mb, chlorophyll, carotenoid contents, and pH values decreased throughout flower development. Meanwhile, flavonoid and total anthocyanin levels first declined and then increased. In Mp, carotenoid, flavonoid, total anthocyanin contents, and pH values gradually decreased during flower development, whereas chlorophyll decreased initially and then increased. Total anthocyanin content in *M. punicea* was significantly higher than in the petals of the other two *Meconopsis* species. In Mi, chlorophyll, carotenoid, flavonoid, and total anthocyanin contents gradually decreased throughout flower development, whereas pH values exhibited the opposite trend. Petals of Mi contained significantly higher flavonoid levels than those of the other two *Meconopsis* species.

Correlation analysis between color indices and pigment contents across the three developmental stages revealed significant relationships among the three species (**Supplementary Table S2**). In Mb and Mp, L* values exhibited significant negative correlations with flavonoid and total anthocyanin contents in both blue and red flowers, indicating that higher pigment concentrations corresponded to darker floral coloration. Moreover, carotenoid content displayed a strong positive correlation with b* values in Mp (*P* < 0.01), suggesting that reduced carotenoid levels might contribute to the decline in b* values. In Mi, b* values showed a strong negative correlation with pigment contents (*P* < 0.01). Furthermore, Mi exhibited significantly higher flavonoid content than the other two *Meconopsis* species, suggesting that specific flavonoid compounds may contribute to yellow petal coloration. Collectively, these findings highlight the pivotal roles of flavonoids and total anthocyanins in determining *Meconopsis* flower coloration.

### Metabolite analysis

**Figure 2:**
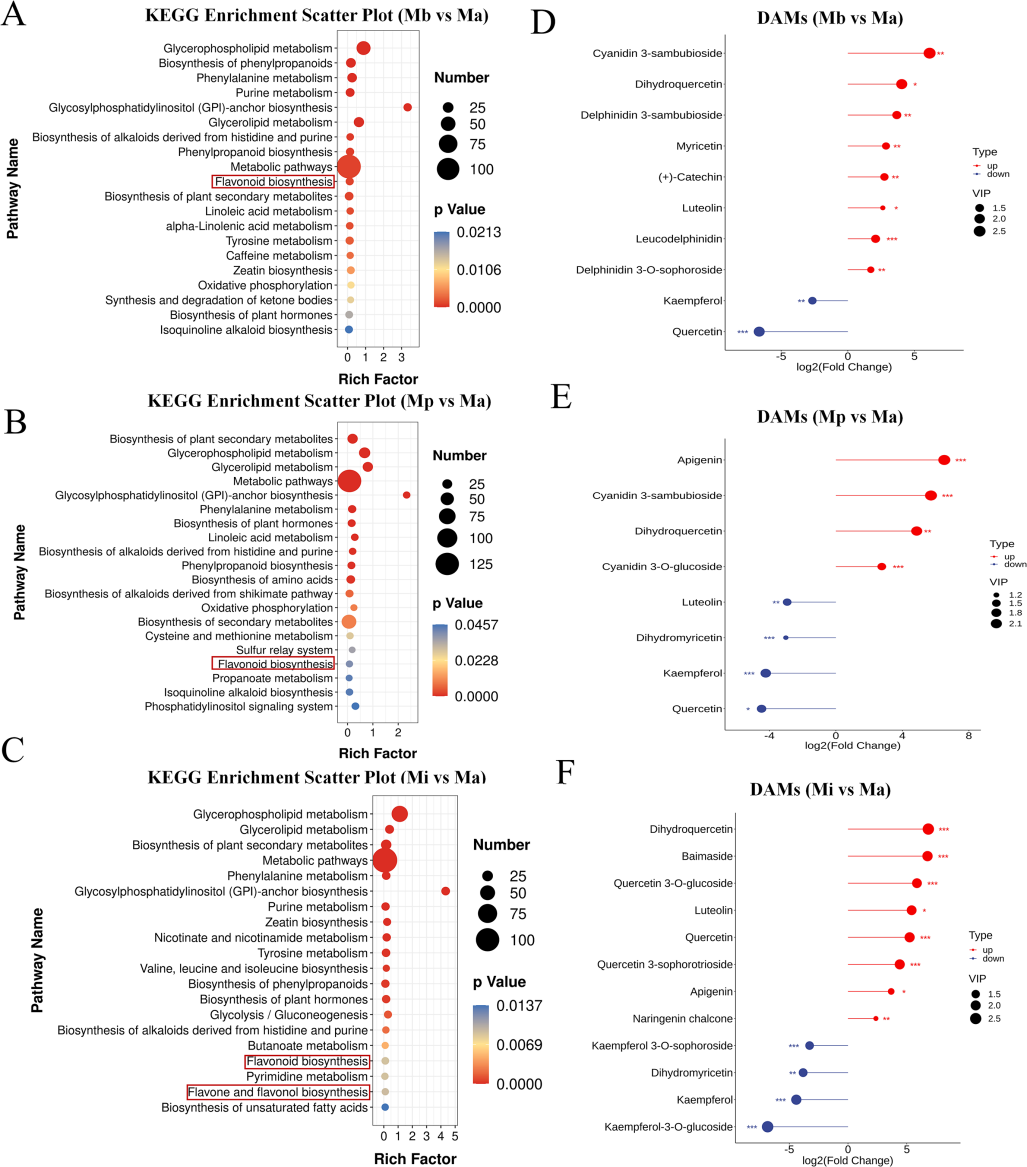
KEGG enrichment and regulatory analysis of DAMs. KEGG enrichment analysis for: **(A)** Mb vs Ma, **(B)** Mp vs Ma, **(C)** Mi vs Ma. Regulatory analysis of DAMs for: **(D)** Mb vs Ma, **(E)** Mp vs Ma, **(F)** Mi vs Ma.

A total of 847 metabolites were identified in the petals of Ma, Mb, Mp and Mi. The classification and quantitative distribution of these metabolites are shown in **Supplementary Figure S2**. The major metabolites included lipids, organic acids, phenolic acids, and flavonoids. Among them, 53 flavonoid metabolites were identified. Flavonols, flavones, and anthocyanins were the most abundant subclasses, accounting for 53%, 19%, and 9% of total flavonoids, respectively. In the significantly enriched pathways (**Figures 2A–C**), using Ma as the control (CK), floral pigment-related pathways in Mb and Mp were mainly enriched in flavonoid biosynthesis (ko00941). Although anthocyanin-related pathways were not significantly enriched, anthocyanin-derived differential metabolites accumulated at high levels in Mb and Mp petals, suggesting a potential role in floral coloration.

In the comparison between Mb and Ma, seven and three differentially accumulated metabolites (DAMs) related to flavonoids and anthocyanins were identified, respectively (**Figure 2D**). Several anthocyanins were significantly upregulated, including cyanidin-3-sambubioside, delphinidin-3-sambubioside, and delphinidin-3-O-sophoroside, with fold changes (FCs) of 70.08, 12.72, and 3.30, respectively. As precursors in anthocyanin biosynthesis, dihydroquercetin and leucodelphinidin were also significantly upregulated, with high fold changes of 16.50 and 4.22, respectively.

In the comparison group between Mp and Ma, six and two DAMs were enriched in the flavonoid and anthocyanin biosynthesis pathways, respectively (**Figure 2E**). The levels of quercetin and kaempferol were downregulated, with fold changes of 22.14 and 18.60. Dihydroquercetin, a key intermediate in anthocyanin glycoside biosynthesis, and anthocyanin showed significant upregulation. Cyanidin-3-sambubioside, dihydroquercetin, and cyanidin-3-O-glucoside exhibited fold changes of 52.60, 29.01, and 6.78, respectively.

In comparison, Mi showed enrichment in flavonoid biosynthesis and flavone/flavonol biosynthesis (ko00944), with eight and four DAMs involved in these pathways, respectively (**Figure 2F**). Four metabolites were downregulated, namely dihydromyricetin, kaempferol, kaempferol-3-O-glucoside, and kaempferol-3-O-sophoroside, with fold changes of 14.11, 21.17, 114.43, and 9.64, respectively. Among the upregulated metabolites, dihydroquercetin had the highest fold change at 114.07, followed by quercetin-3-O-sophoroside at 108.31. Naringenin chalcone, an intermediate in flavonol biosynthesis, also showed an upregulation trend, with a fold change of 5.18.

### Transcriptome analysis of the petals of three *Meconopsis* varieties

**Figure 3:**
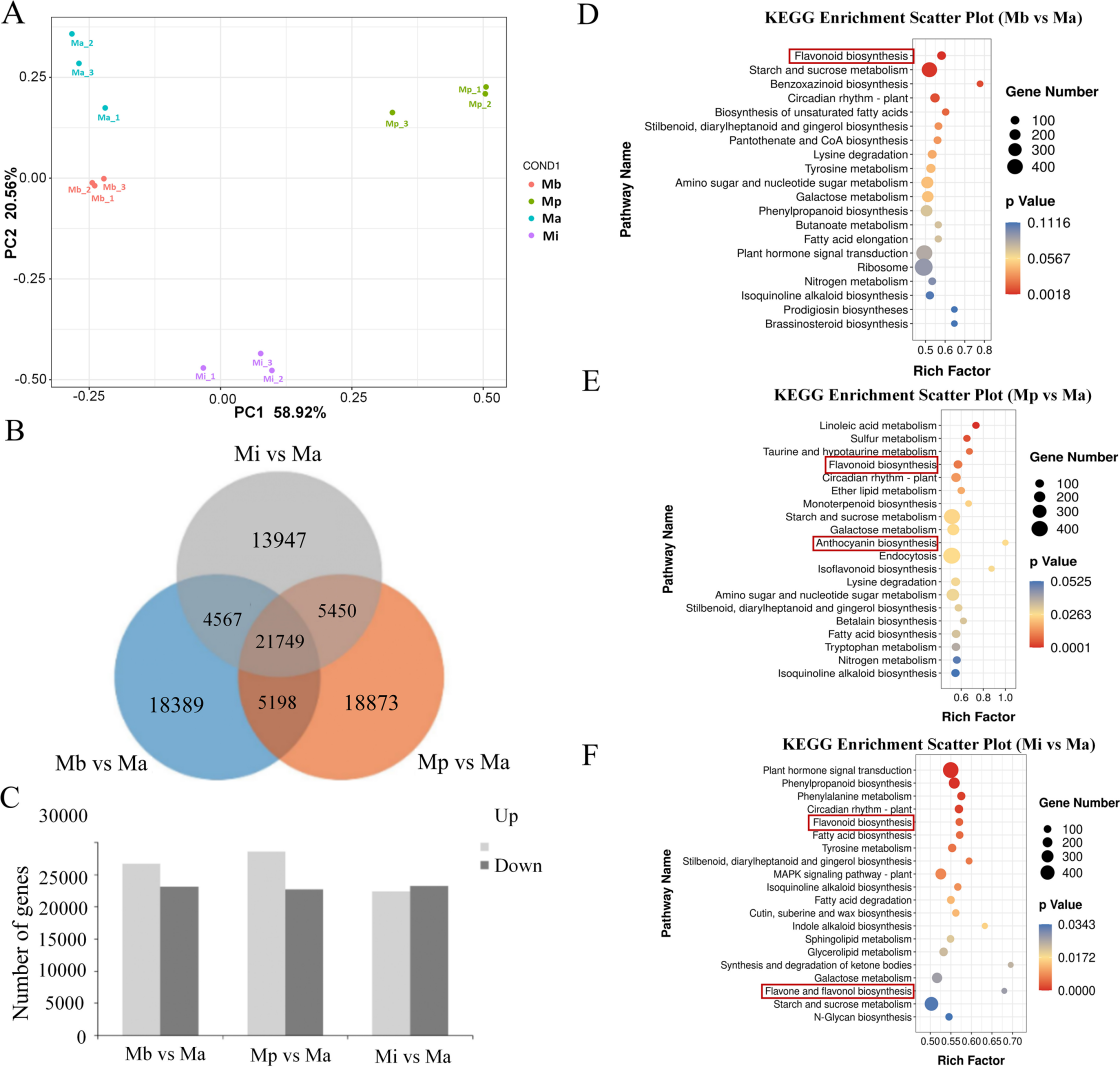
Preliminary analysis of transcriptome data. **(A)** PCA score plot of mass spectrometry data for each sample. **(B)** Venn diagrams of DEGs across comparison groups. **(C)** Statistics of DEGs numbers. KEGG enrichment analysis for: **(D)** Mb vs Ma, **(E)** Mp vs Ma, **(F)** Mi vs Ma.

RNA-sequencing (RNA-seq) was used to analyze gene expression profile changes and understand the molecular mechanisms underlying different flower colors. As *Meconopsis* lacks a reference genome sequence, the transcript sequences assembled by Trinity were utilized as the reference for subsequent analyses. With three biological replicates, a total of 498,759,980 clean reads (representing 73.05 GB of clean data) were generated from 12 petal samples (**Supplementary Table S3**). In total, 569,449 transcripts and 122,876 unigenes were obtained (**Supplementary Table S4**). To annotate these unigenes, sequence comparisons were performed against multiple databases, including GO (34.37% of unigenes), KEGG (14.61%), Pfam (27.20%), Swiss-Prot (27.67%), eggNOG (38.44%), NR (51.52%), and transcription factors (TF, 1.99%) (**Supplementary Table S5**). PCA score plot of mass spectrometry data for each sample confirmed that the experimental procedures were reproducible and suitable for subsequent analyses (**Figure 3A**).

Using Ma as a control, differentially expressed genes (DEGs) were selected based on the criteria of |log_2_Fold Change| ≥ 1 and FDR< 0.05. Venn diagrams were constructed for the differentially expressed genes in each comparison group (**Figure 3B**). Furthermore, the total number of DEGs, upregulated genes, and downregulated genes was calculated for each comparison (**Figure 3C**). In the Mb vs Ma comparison group, 26,738 upregulated and 23,165 downregulated DEGs were observed. In the Mp vs Ma comparison, the numbers of upregulated and downregulated DEGs were 28,552 and 22,718, respectively. The Mi vs Ma comparison identified 45,613 DEGs, with 22,369 upregulated and 23,244 downregulated, respectively.

With Ma as the control, the KEGG enrichment analysis of DEGs in the three comparison groups is shown in **Figures 3D–F**. DEGs were significantly enriched in pathways such as flavonoid biosynthesis (ko00941), circadian rhythm (ko04712), and plant hormone signal transduction (ko04075) across all three comparison groups. DEGs in Mp were significantly enriched in the anthocyanin biosynthesis pathway. In contrast, the comparison between Mi and Ma showed specific enrichment in the flavone and flavonol biosynthesis pathway (ko00944). These pathways, including flavonoid biosynthesis (ko00941), anthocyanin biosynthesis (ko00942), and flavonol biosynthesis (ko00944), were recognized as critical for regulating petal pigmentation.

### DEGs involved in flavonoid biosynthesis pathways

**Figure 4:**
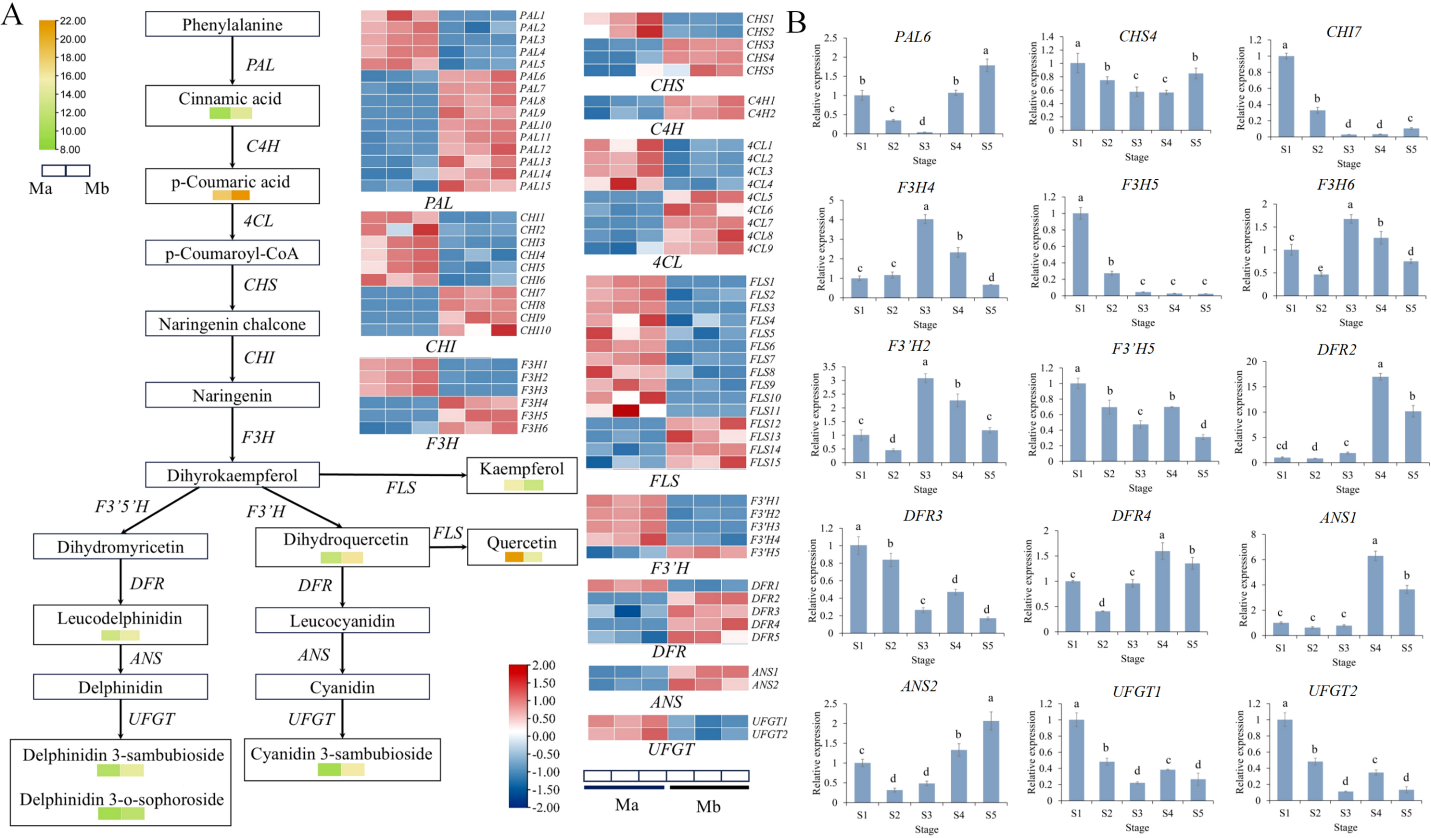
Heat map of DEGs in the flavonoid biosynthesis pathway in Mb. **(A)** The arrows indicate the synthesis steps. The heatmap with yellow-green color scheme represents the relative content of DAMs. The heatmap with red-blue color scheme indicates DEGs. **(B)** Relative expression of key structural genes of Mb at five stages. Different letters (a, b, c, d, e) indicate significant differences at different sampling stages (*p* < 0.05).

**Figure 5:**
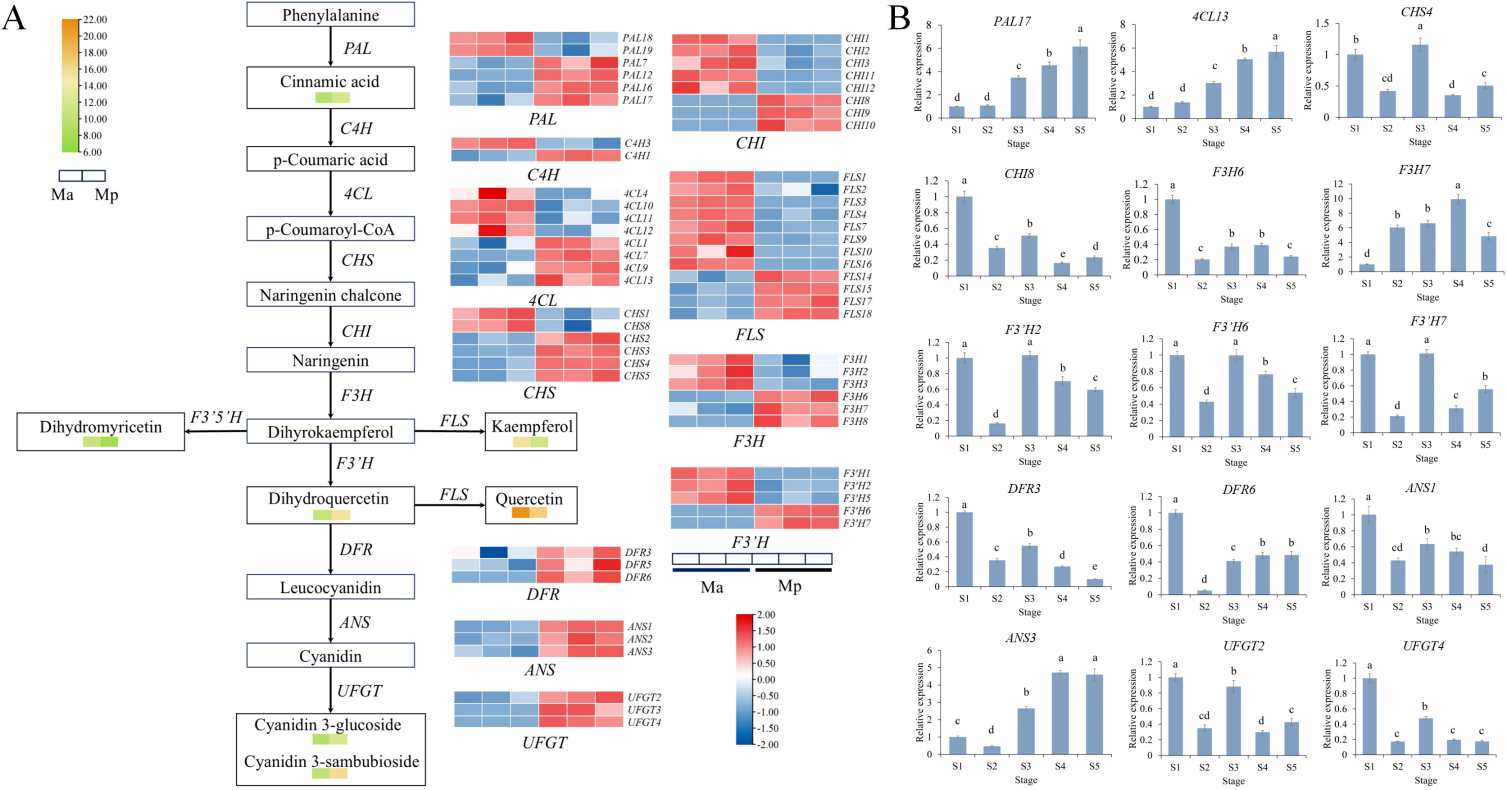
Heat map of DEGs in the flavonoid biosynthesis pathway in Mp. **(A)** The arrows indicate the synthesis steps. The heatmap with yellow-green color scheme represents the relative content of DAMs. The heatmap with red-blue color scheme indicates DEGs. **(B)** Relative expression of key structural genes of Mp at five stages. Different letters (a, b, c, d, e) indicate significant differences at different sampling stages (*p* < 0.05).

**Figure 6:**
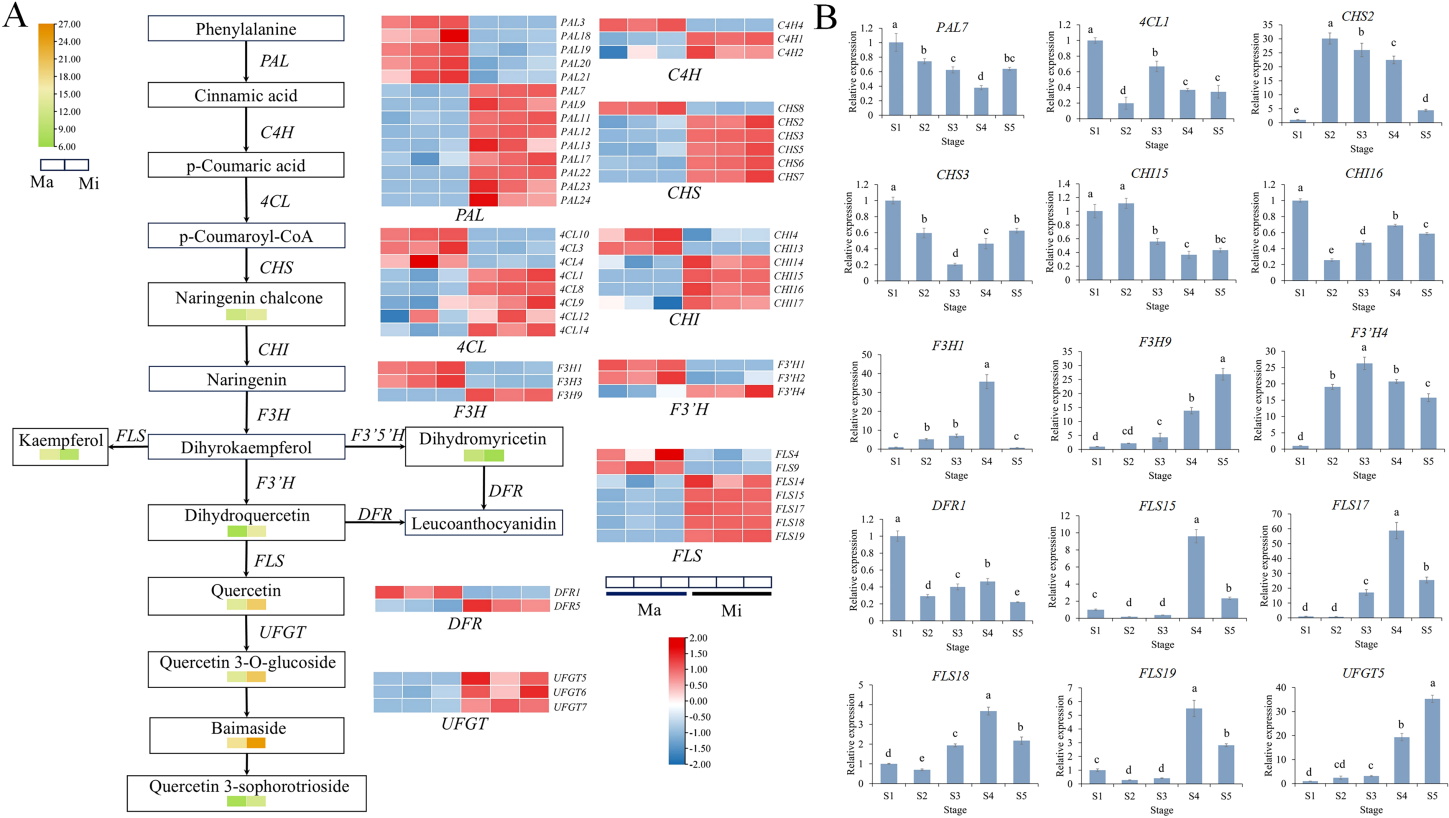
**(A)** Heat map of DEGs Flavonoid and flavonol biosynthesis pathway in Mi. The arrows indicate the synthesis steps. The heatmap with yellow-green color scheme represents the relative content of DAMs. The heatmap with red-blue color scheme indicates DEGs. **(B)** Relative expression of key structural genes of Mi at five stages. Different letters (a, b, c, d, e) indicate significant differences at different sampling stages (*p* < 0.05).

Combined metabolomic and transcriptomic analyses revealed that Mb, Mp and Mi was significantly enriched in the flavonoid biosynthesis pathway. The flavone and flavonol biosynthesis pathway was also identified in Mi. The unigenes involved in the flavonoid biosynthesis pathway were screened from the RNA-seq data set. The expression profiles of flavonoid biosynthesis pathway structural genes in the different colored petals based on their TPM values were illustrated using a heatmap analysis. Building upon existing research in the flavonoid biosynthesis pathway, a heatmap of DEGs of Mb, Mp and Mi has been generated (**Figures 4A**, **5A** and **6A**) (Qu et al., 2022; Chen et al., 2024; Wang et al., 2024; Kong et al., 2025; Ni et al., 2025). qRT-PCR analysis was performed for 15 genes to explore the expression patterns of key DEGs at five stages of flower development in three species (**Figures 4B**, **5B**, **6B**). The qRT-PCR results of the DEGs showed the same trend as the RNA-Seq results, indicating the high reliability of the data obtained by transcriptome sequencing (**Supplementary Figures S5**–**S7**). Correlation analyses were also conducted between the relative expression levels of key genes at three floral developmental stages and the contents of flavonoids and total anthocyanins.

### Flavonoid biosynthesis pathway in Mb

A total of 76 structural genes from 11 gene families involved in anthocyanin biosynthesis were identified in the Mb genome. Among these, ten *PALs*, two *C4Hs*, five *4CLs*, three *CHSs*, four *CHI*, three *F3Hs*, one *F3’Hs*, four *DFRs*, and two *ANSs* genes showed significant upregulation in Mb. Among the late biosynthetic genes, *DFR2* and *DFR4* were not expressed in Ma but were highly expressed in Mb. *ANS1* and *ANS2* were upregulated by 5.58- and 5.00-fold, respectively. In contrast, *UFGT* genes were expressed at low levels in Mb, despite the significant accumulation of their catalytic products, cyanidin glycoside and delphinidin glycoside. To further examine their temporal expression dynamics, gene expression in Mb petals was analyzed across five floral developmental stages. *UFGT1* and *UFGT2* showed peak expression at early bud stage (S1). This suggests that *UFGT* activity is temporally regulated — functioning o during early bud differentiation to promote anthocyanin accumulation, followed by reduced expression as floral organs mature and pigmentation stabilizes (**Figure 4B**). Pearson correlation analysis between gene expression and anthocyanin content across developmental stages revealed that *PAL6*, *F3’H5*, *DFR3*, *ANS2*, *UFGT1*, and *UFGT2* were significantly positively correlated with total anthocyanin content (**Supplementary Table S9**), suggesting their potential roles in promoting anthocyanin biosynthesis and petal coloration in Mb.

### Flavonoid biosynthesis pathway in Mp

A total of 34 differentially upregulated genes and 28 differentially downregulated genes were identified in Mp vs Ma. Among these, four *PALs*, one *C4H*, four *4CLs*, four *CHSs*, three *CHIs*, three *F3Hs*, two *F3’Hs*, three *DFRs*, and three *ANSs* showed significant upregulation in Mp, thereby positively regulating the synthesis of the pigmented compound cyanidin glycosides. As *F3’H*, *DFR*, and *FLS* were located at the branching positions of anthocyanin biosynthesis mentioned above, their expression specificity deserved special attention to unveil the molecular mechanisms underlying spatial anthocyanin biosynthesis in Mp. Dihydromyricetin and kaempferol, catalyzed by *F3’5’H* and *FLS* respectively, showed significant decreases, whereas dihydroquercetin derived from *F3’H* accumulated substantially in Mp. Consequently, metabolic flux was redirected toward anthocyanin biosynthesis.

To gain deeper insights into the spatiotemporal expression patterns of these genes, the relative expression levels in Mp petals were examined at five successive flowering stages. Most of the genes’ expression levels exhibited a pattern of first decreasing, then increasing, and finally decreasing again. These genes showed high expression at S1 stage and were significantly downregulated at S2 stage (**Figure 5B**). Pearson’s correlation analysis showed that the expressions of *ANS1*, *DFR3*, *DFR6*, *F3H6*, *F3’H2*, *F3’H6*, *F3’H7*, *CHI8*, *CHS4*, *UFGT2* and *UFGT4* were significantly positively correlated with total anthocyanin accumulation (**Supplementary Table S10**), suggesting that these genes may promote anthocyanin biosynthesis.

### Flavonoid and flavonol biosynthesis pathway in Mi

A total of 36 up- and 19 downregulated genes were detected in Mi vs Ma. The upregulated genes included nine *PALs*, two *C4Hs*, five *4CLs*, five *CHSs*, four *CHIs*, one *F3H*, one *F3’H*, and three *FLSs* genes that showed significant upregulation in Mi, thereby positively regulating the synthesis of quercetin glycosides. *F3’5’H*, *FLS*, *DFR*, and *F3’H* competed for the substrate dihydrokaempferol. *DFR1* showed low expression levels in Mi, while *F3’H4* had a relatively large log_2_FC of 2.88. *FLS14*, *FLS17*, and *FLS18* were upregulated by 4.12-fold, 11.94-fold, and 12.17-fold, respectively. The strong expression of *FLS* directed the metabolic flux toward quercetin, which was further glycosylated into quercetin glycosides.

To further elucidate the temporal expression dynamics of these genes, their expression patterns were analyzed across five floral developmental stages in Mi. Expression levels of *F3H1*, *F3H9*, *FLS17*, *FLS18*, and *UFGT5* showed an upward trend during floral development, with low expression from S1 to S3 stage and high expression at S4 stage (**Figure 6B**). In addition, these genes showed a significant negative correlation with flavonoid content. (**Supplementary Table S11**). Given that the yellow color of Mi petals was primarily determined by quercetin derivatives among flavonoids, the expression of these genes showed a synchronous trend with the gradual increase in the yellowness b* value during the flowering period. It is thus hypothesized that these genes may exert a critical function during the yellow flower development of Mi.

### Analysis of transcription factors

**Figure 7:**
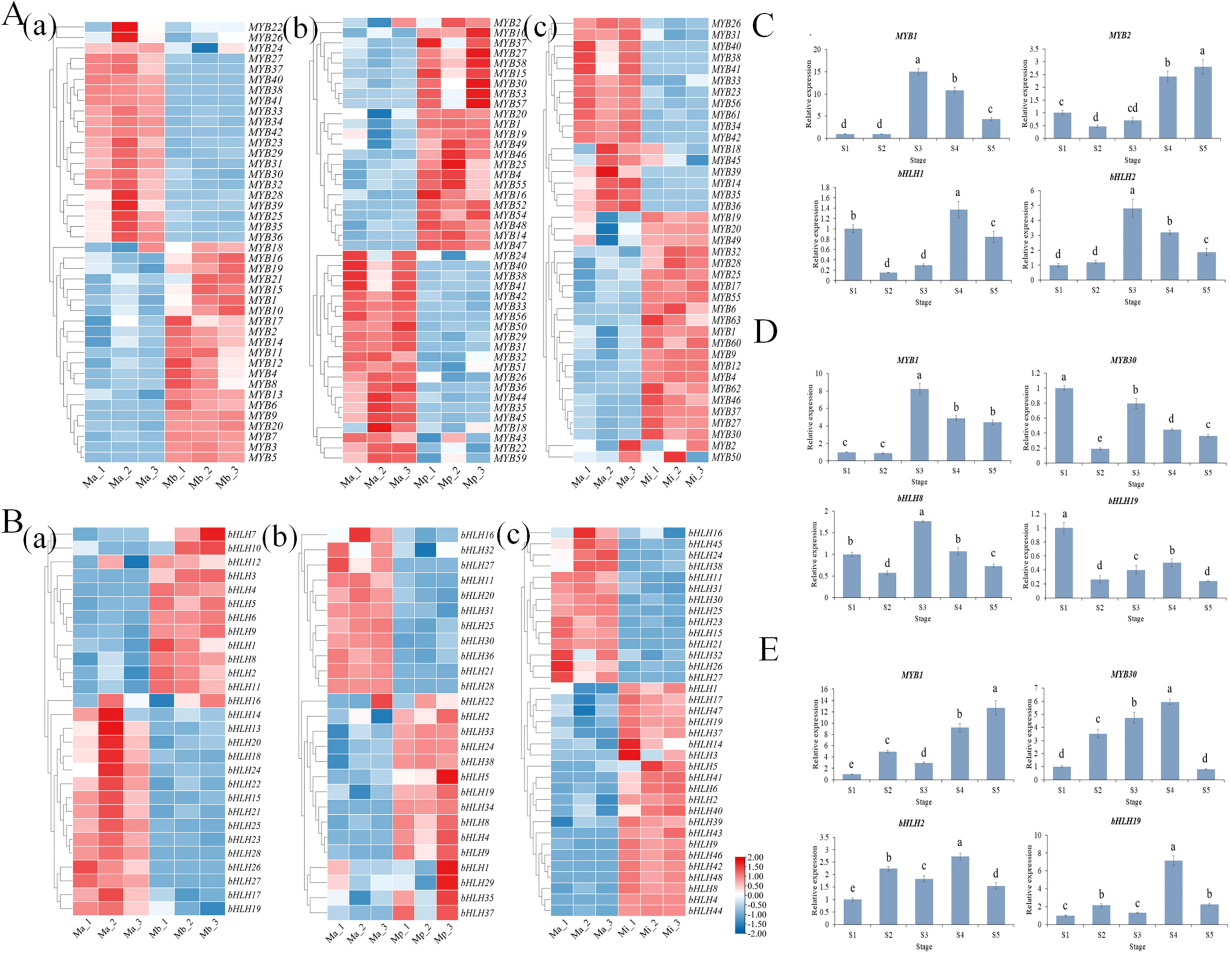
Expression pattern analysis of differentially expressed *MYBs* and *bHLHs.***(A)** Heatmap of MYB transcription factor expression levels. **(B)** Heatmap of bHLH transcription factor expression levels. **(a)**: Mb vs Ma, **(b)**: Mp vs Ma, **(c)**: Mi vs Ma. Relative expression analysis of *MYBs* and *bHLHs* in three *Meconopsis* species across five stages: **(C)** Mb; **(D)** Mp; **(E)** Mi. Different letters (a, b, c, d, e) indicate significant differences at different sampling stages (p< 0.05).

Transcription factors (TFs) were predicted using the PlantTFDB website and integrated with transcriptome annotation, leading to the identification of a total of 54 classes of differentially expressed TFs. The top five families with the highest number of members are shown in **Supplementary Figure S4**, including the *ERF*, *MYB*, *bHLH* families. *MYB* and *bHLH* were the transcription factor families most frequently involved in flower color research. The heatmaps of genes encoding these two transcription factor families were shown (**Figures 7A, B**). Comparative analysis using Ma as the control revealed differential expression of *MYB* and *bHLH* genes across the three species: Mb (42 *MYBs*, 27 *bHLHs*), Mp (44 *MYBs*, 26 *bHLHs*), and Mi (38 *MYBs*, 36 *bHLHs*). In each of the three *Meconopsis* species, two differentially expressed *MYB* and two *bHLH* genes were identified. Their relative expression levels were measured across five floral developmental stages (**Figures 7C–E**). Correlation analyses were performed between gene expression and the contents of flavonoids and total anthocyanins (**Supplementary Tables S9**–**S11**). Results indicated that *MbbHLH1*, *MbMYB30* and *MpbHLH19* exhibited significant positive correlations with total anthocyanin content, implying a positive regulatory role in anthocyanin synthesis and floral pigmentation. *MiMYB1*, *MiMYB30*, *MibHLH2* and *MibHLH19* were negatively correlated with flavonoid content, while their expression trends matched the progressive elevation of yellowness b* value during flowering, which suggests their critical function in yellow flower development.

## Discussion

### Floral pigments and pH regulate petal coloration in *Meconopsis*

The results of this study revealed that petal coloration in *Meconopsis* is primarily governed by variations in pigment composition and petal pH. In *M. balangensis* (Mb), the significant correlation between L* and b* values, but not a* values, indicates that lightness is mainly affected by petal blueness. The significant positive correlation among the L* value, b* value, flavonoids and total anthocyanins indicates that these substances are the major determinants of blue petal pigmentation, which is consistent with the findings of previous studies (Zan et al., 2024). In *Primula vulgaris*, *Petunia hybrida* and *Pueraria lobata*, blue or purple flower morphs consistently exhibit higher petal pH values than red morphs (Quattrocchio et al., 2006; Tatsuzawa et al., 2017; Zhai et al., 2020). The petal pH of *M. balangensis* is higher than that of *M. punicea*, potentially due to the stability of anthocyanins, which retain red coloration at lower pH but shift toward blue and become unstable at higher vacuolar pH levels (Yoshida et al., 2010). The blue pigment in *M. grandis* is likely a novel type of metal complex pigment. Researchers successfully reproduced a comparable blue hue by combining anthocyanins, flavonols, and metal ions in a buffered solution at pH 5.0 (Yoshida et al., 2006; Qu et al., 2022). Given the critical role of petal pH in blue coloration in *Meconopsis*, future research should focus on elucidating the regulatory mechanisms of pH-related genes.

In *M. punicea* (Mp), the high anthocyanin content was strongly and positively correlated with the a* (redness) value, consistent with earlier studies in *Paeonia suffruticosa* (Luo et al., 2021). These results indicate that total anthocyanin concentration is the primary factor contributing to red coloration. Anthocyanins display stable red coloration under low-pH conditions, and changes in their stability directly contribute to floral color variation (Tanaka et al., 1998). In *M. punicea*, a significant positive correlation was observed among a*, pH, and total anthocyanin content, suggesting that pH may affect red coloration by modulating anthocyanin stability (Yu et al., 2024). In *M. punicea*, carotenoid content was strongly positively correlated with the b* (yellowness) value, indicating that reduced carotenoid levels may lower b* values, consistent with observations in *Edgeworthia chrysantha* (Zhou et al., 2023). Variations in carotenoid content may reduce petal yellowness and result in subtle changes in floral coloration in *M. punicea*. Previous studies on *M. punicea* suggest that carotenoids may play a regulatory role in flower coloration (Qu et al., 2022).

In *M. integrifolia* (Mi), lightness (L*) was positively correlated with flavonoid content, while yellowness (b*) showed a significant negative correlation (*P* < 0.01). Flavonoid content was significantly higher than that in the other two species, whereas anthocyanin content was comparatively lower. Flavones and flavonols have been shown to impart white to pale yellow coloration to petals (Lin et al., 2021). Quercetin derivatives, a class of flavonols, have been reported as the major yellow pigments responsible for the floral coloration in *Camellia nitidissima* and *Osmanthus fragrans* (Zhou et al., 2013; Zou et al., 2017). Therefore, the high accumulation of specific flavones and flavonols may contribute to the yellow petal coloration in this species. The petal color of *Camellia reticulata* deepens progressively, accompanied by a gradual decrease in pH (Xue et al., 2015). Compared with *M. balangensis* and *M. punicea*, the petals of the light-colored species *M. integrifolia* exhibit a higher pH, which may enhance the stability of yellow pigments (Yoshida et al., 2010).

### Flavonoids determine color diversification among *Meconopsis* species

Flavonoids were identified as the central pigment group responsible for color diversity in *Meconopsis*. In blue flowers of *M. balangensis*, high accumulation of cyanidin-3-sambubioside, delphinidin-3-sambubioside, and delphinidin-3-O-sophoroside was detected, consistent with reports that delphinidin derivatives confer blue coloration in *M. horridula* and *M. betonicifolia* (Tanaka et al., 2001). Flavones and flavonols act as copigments with chromogenic anthocyanins, thus facilitating the bluing or deepening of floral coloration (Mizuno et al., 2013). The copigment-induced enhancement of blue coloration has been documented in *M. horridula*, *M. betonicifolia*, *Torenia fournieri* and *Dahlia variabilis* (Aida et al., 2000; Tanaka et al., 2001; Ayumi et al., 2013). Luteolin (a flavone) and myricetin (a flavonol), both significantly accumulated in *M. balangensis*, may function as copigments that enhance the coloration effect of delphinidin glycosides, thereby contributing to the blue hue of the petals.

Red petal coloration in *M. punicea* was mainly associated with the accumulation of cyanidin glycosides, especially cyanidin-3-glucoside and cyanidin-3-sambubioside, in agreement with findings in *Camellia japonica* and *Brassica napus* (Li et al., 2019; Ye et al., 2022). In contrast, yellow pigmentation in *M. integrifolia* resulted from the presence of quercetin derivatives. Quercetin-derived compounds have been identified as the yellow pigments responsible for floral coloration in a variety of plant species, such as *Camellia nitidissima* and *Paeonia* spp (Zhou et al., 2013; Yang et al., 2020).

### Transcriptional regulation of flavonoid biosynthesis underlies color differentiation

High expression levels of key genes in the flavonoid metabolite pathway promote the production of different floral pigments, which has been investigated in *Lysimachia arvensis*, *Camellia sinensis*, and *Rosa multiflora* (Luo et al., 2015; Kipkoech et al., 2021; Mercedes et al., 2021). Integrated transcriptomic and metabolomic analyses revealed that the three *Meconopsis* species share a conserved flavonoid biosynthetic framework, yet differ in the regulation of key structural genes and transcription factors. In *M. balangensis* and *M. punicea*, high expression of *F3Hs*, *F3’Hs, DFRs* and *ANSs* directed metabolic flux toward anthocyanin biosynthesis, producing blue and red pigments. In *Paeonia lactiflora*, key structural genes such as *ANS*, *DFR*, *F3H* and *UFGT* are highly expressed at the early flowering stage and decrease at full bloom, mirroring the trend of total anthocyanin content (Zhao et al., 2012). This expression pattern resembles that of *UFGT* in *M. balangensis* at different floral developmental stages observed in this study. High expression levels of *FLS* and *UFGT* promoted flavonol biosynthesis, leading to quercetin accumulation and yellow pigmentation in *M. integrifolia*, and these observations match earlier findings in *M. integrifolia* and *Dasiphora* (Chen et al., 2024; Tian et al., 2025).

The MYB, bHLH, and WD40 transcription factors form the MBW complex, which can activate or repress the expression of structural genes, thereby regulating flavonoid metabolism (Hichri et al., 2011; Nakatsuka et al., 2012). Studies in various species, including *Aglaonema commutatum*, *Dendrobium officinale*, and *Gentiana triflora*, have shown that *MYBs* and *bHLHs* bind to the promoters of structural genes such as *CHSs* and *DFRs*, either independently or synergistically, to activate gene expression and enhance anthocyanin biosynthesis (Nakatsuka et al., 2012; Li et al., 2022; Yang et al., 2023). Notably, their overexpression can also repress *DFRs* expression and reduce anthocyanin accumulation (Filyushin et al., 2023). In this study, *MYBs* and *bHLHs* showed significant correlations with the expression of key structural genes (**Supplementary Tables S9**-**S11**). In *M. balangensis* and *M. punicea*, *MYBs* and *bHLHs* may promote anthocyanin accumulation by upregulating the expression of *F3Hs*, *F3’Hs*, *DFRs*, and *ANSs*. In contrast, in *M. integrifolia*, these transcription factors may facilitate flavonol accumulation by enhancing the expression of *CHS2*, *F3H1*, *F3H9*, *F3’H4*, *FLS17*, *FLS18*, and *UFGT5*, or by repressing *DFR1* expression, thereby contributing to yellow flower pigmentation.

**Figure 8:**
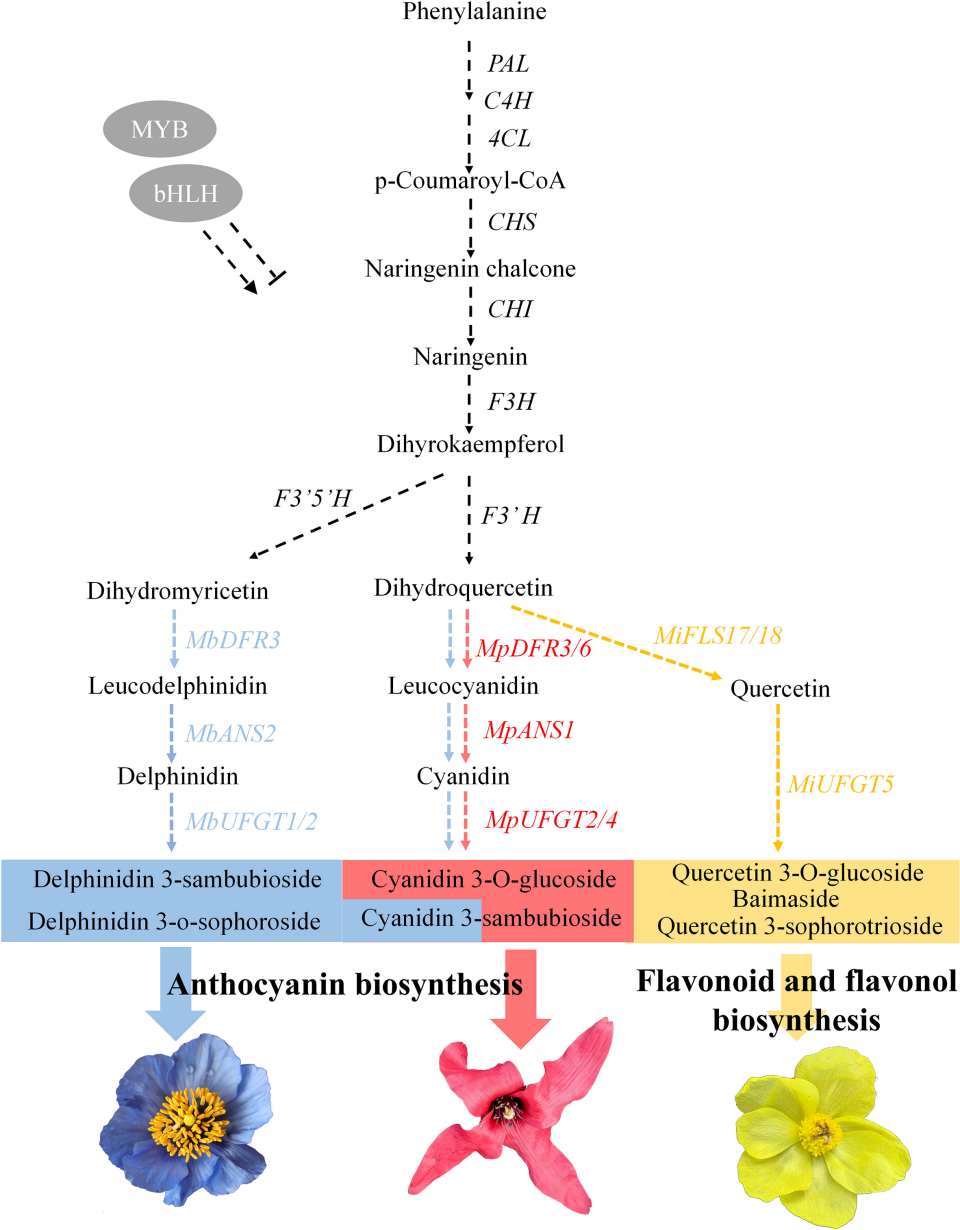
Schematic of molecular regulation of flower color formation in three *Meconopsis* species. Black arrows indicate the upstream metabolic pathways shared by the three *Meconopsis* species. Blue, red, and yellow arrows represent the downstream pathways for the synthesis of blue, red, and yellow flowers, respectively. Blue, red and yellow rectangles represent the pigmented substances corresponding to the flower color, respectively. Cyanidin 3-sambubioside is a common pigment in blue and red flowers.

A regulatory network diagram depicting the molecular mechanisms governing pigmentation in these three species was developed (**Figure 8**). The formation of the three flower colors in *Meconopsis* is fundamentally driven by the flavonoid metabolic pathway. The coordinated action of structural genes (*DFR*, *FLS*, *UFGT*) and transcription factors (MYB, bHLH) shapes the biosynthetic direction toward anthocyanin- or flavonol-dominated pigmentation. This integrated understanding not only elucidates the molecular basis of petal color diversification in *Meconopsis* but also provides genetic targets for future molecular breeding and pigment engineering in alpine ornamental plants.

## Data Availability

The datasets presented in this study can be found in online repositories. The names of the repository/repositories and accession number(s) can be found in the National Center for Biotechnology Information (NCBI) BioProject database under accession number PRJNA1358786.

## Supplementary material

Supplementary Figure 1 Scanning electron microscopy of petals in full bloom stage.

Supplementary Figure 2 Classification and Quantity Statistics of Identified Metabolites.

Supplementary Figure 3 GO enrichment analysis of DEGs.

Supplementary Figure 4 Differential Transcription Factor Classification Chart.

Supplementary Figure 5 Validation of qRT-PCR of Mb.

Supplementary Figure 6 Validation of qRT-PCR of Mp.

Supplementary Figure 7 Validation of qRT-PCR of Mi.

## References

[B1] Aida R. Yoshida K. Kondo T. Kishimoto S. Shibata M. (2000). Copigmentation gives bluer flowers on transgenic torenia plants with the antisense dihydroflavonol-4-reductase gene. Plant Sci. 160, 49–56. doi: 10.1016/50168-9452(00)00364-2, PMID: 11164576

[B2] An J. Yao J. Xu R. You C. Wang X. Hao Y. (2018). Apple bZIP transcription factor *MdbZIP44* regulates ABA-promoted anthocyanin accumulation. Plant Cell Environ. 41, 2678–2692. doi: 10.1111/pce.13393, PMID: 29940702

[B3] Antonio G. Zhao M. Leavitt J. M. Lloyd A. M. (2008). Regulation of the anthocyanin biosynthetic pathway by the TTG1/bHLH/Myb transcriptional complex in Arabidopsis seedlings. Plant J.: Cell Mol. Biol. 53, 814–827. doi: 10.1111/j.1365-313X.2007.03373.x, PMID: 18036197

[B4] Ayumi D. Sho O. Munetaka H. Fumi T. Motoaki D. (2013). Endogenous post-transcriptional gene silencing of flavone synthase resulting in high accumulation of anthocyanins in black *dahlia cultivars*. Planta 237, 1325–1335. doi: 10.1007/s00425-013-1848-6, PMID: 23389674

[B5] Chen X. Luo J. Wang F. Li T. Qu Y. (2024). Excavation of key genes for yellow flower formation in *Meconopsis integrifolia*. Acta Agric. Zhejiangensis 36, 811–824. doi: 10.3969/j.issn.1004-1524.20230535

[B6] Chen Z. Yang Z. Wang G. (2023). Analysis of germplasm resources and introduction conservation of *meconopsis*. Seed Sci. Technol. 41, 124–126. doi: 10.19904/j.cnki.cn14-1160/s.2023.09.042

[B7] Filyushin M. Shchennikova A. Kochieva E. (2023). Coexpression of structural and regulatory genes of the flavonoid pathway reveals the characteristics of anthocyanin biosynthesis in eggplant organs (*Solanum melongena* L.). Russian J. Plant Physiol. 70, 27. doi: 10.1134/S1021443722603147

[B8] Fraser C. M. Chapple C. (2011). The phenylpropanoid pathway in *Arabidopsis*. Arabidopsis Book 9, e0152–e0152. doi: 10.1199/tab.0152, PMID: 22303276 PMC3268504

[B9] Gonnet J.-F. (2001). Colour effects of co-pigmentation of anthocyanin revisited—3. A further description using CIELAB differences and assessment of matched colours using the CMC model. Food Chem. 75, 473–485. doi: 10.1016/S0308-8146(98)00053-3

[B10] Grotewold E. (2006). The genetics and biochemistry of floral pigments. Annu. Rev. Plant Biol. 57, 761–780. doi: 10.1146/annurev.arplant.57.032905.105248, PMID: 16669781

[B11] Gu C. Hong S. Wang J. Shang L. Zhang G. Zhao Y. . (2024). Identification and expression analysis of the bZIP and WRKY gene families during anthocyanins biosynthesis in *Lagerstroemia indica* L. Hortic. Environ. Biotechnol. 65, 169–180. doi: 10.1007/s13580-023-00551-w

[B12] He N. Yang Y. Jiang H. Peng Q. Wei Z. Zhang W. (2021). Correlations among Petal Color, pH, and Epidermal Cell Morphology of *Ornamental Crabapple*. Fujian J. Agric. Sci. 36, 1025–1032. doi: 10.19303/j.issn.1008-0384.2021.09.005

[B13] Hichri I. Barrieu F. Bogs J. Kappel C. Delrot S. Lauvergeat V. (2011). Recent advances in the transcriptional regulation of the flavonoid biosynthetic pathway. J. Exp. Bot. 62, 2465–2483. doi: 10.1093/jxb/erq442, PMID: 21278228

[B14] Iwashina T. (2015). Contribution to flower colors of flavonoids including anthocyanins: A review. Natural Prod. Commun. 10, 529–544. doi: 10.1177/1934578X1501000335, PMID: 25924543

[B15] Jin X. Jin X. Huang H. Wang L. Sun Y. Dai S. (2016). Transcriptomics and metabolite analysis reveals the molecular mechanism of anthocyanin biosynthesis branch pathway in different *Senecio cruentus* cultivars. Front. Plant Sci. 7. doi: 10.3389/fpls.2016.01307, PMID: 27656188 PMC5012328

[B16] Kipkoech M. T. Mamta M. Romit S. Kumar S. R. (2021). Transcriptional analysis reveals key insights into seasonal induced anthocyanin degradation and leaf color transition in purple tea (*Camellia sinensis* (L.) O. Kuntze). Sci. Rep. 11, 1244–1244. doi: 10.1038/s41598-020-80437-4, PMID: 33441891 PMC7806957

[B17] Koes R. Verweij W. Quattrocchio F. (2005). Flavonoids: a colorful model for the regulation and evolution of biochemical pathways. Trends Plant Sci. 10, 236–242. doi: 10.1016/j.tplants.2005.03.002, PMID: 15882656

[B18] Kong D. Jiang T. Zhao J. Yang C. Li S. Shan Q. . (2025). Genome-wide identification and analysis of petal color related MYB transcription factor gene in *Gerbera hybrida*. Ind. Crops Prod. 232, 121284. doi: 10.1016/j.indcrop.2025.121284

[B19] Li J. Wu K. Li L. Ma G. Fang L. Zeng S. (2022). *AcMYB1* interacts with *AcbHLH1* to regulate anthocyanin biosynthesis in *Aglaonema commutatum*. Front. Plant Sci. 13. doi: 10.3389/fpls.2022.886313, PMID: 35928704 PMC9344012

[B20] Lin Q. Liu T. Liu J. Cai M. Cheng T. Wang J. . (2021). Flavonoids composition and content in petals of *lagerstroemia* and *heimia* species and cultivars. Acta Hortic. Sin. 40, 1956–1968. doi: 10.16420/j.issn.0513-353x.2021-0408

[B21] Li X. Wang J. Yin H. Fan Z. Li J. (2019). Variation of flower colors and their relationships with anthocyanins in cultivars of *Camellia japonica*. J. Ecol. Rural Environ. 35, 1307–1313. doi: 10.19741/j.issn.1673-4831.2019.0043

[B22] Liu J. Wang S. Yang X. Chang K. Zhang H. (2025). The regulatory role of bHLH transcription factors in plant anthocyanin biosynthesis. J. Plant Genet. Resour. 26, 844–853. doi: 10.13430/j.cnki.jpgr.20240923002

[B23] Liu W. Feng Y. Yu S. Fan Z. Li X. Li J. . (2021). The flavonoid biosynthesis network in plants. Int. J. Mol. Sci. 22, 12824–12824. doi: 10.3390/ijms222312824, PMID: 34884627 PMC8657439

[B24] Luo P. Ning G. Wang Z. Shen Y. Jin H. Li P. . (2015). Disequilibrium of flavonol synthase and dihydroflavonol-4-reductase expression associated tightly to white vs. Red color flower formation in plants. Front. Plant Sci. 6. doi: 10.3389/fpls.2015.01257, PMID: 26793227 PMC4710699

[B25] Luo X. Sun D. Wang S. Luo S. Fu Y. Niu L. . (2021). Integrating full-length transcriptomics and metabolomics reveals the regulatory mechanisms underlying yellow pigmentation in tree peony (*Paeonia suffruticosa* Andr.) flowers. Hortic. Res. 8, 235–235. doi: 10.1038/s41438-021-00666-0, PMID: 34719694 PMC8558324

[B26] Martin C. Prescott A. Mackay S. Bartlett J. Vrijlandt E. (1991). Control of anthocyanin biosynthesis in flowers of *Antirrhinum majus*. Plant J. 1, 37–49. doi: 10.1111/j.1365-313X.1991.00037.x, PMID: 1844879

[B27] Masahiro N. Akiko H. Takuya T. Shota U. Hideyuki T. (2024). Flower color modification in *Torenia fournieri* by genetic engineering of betacyanin pigments. BMC Plant Biol. 24, 614. doi: 10.1186/s12870-024-05284-1, PMID: 38937670 PMC11210153

[B28] Mercedes S. C. Javier J. L. F. Eduardo N. Montserrat A. L. O. P. J. R. C. F. . (2021). Changes at a Critical Branchpoint in the Anthocyanin Biosynthetic Pathway Underlie the Blue to Orange Flower Color Transition in *Lysimachia arvensis*. Front. Plant Sci. 12. doi: 10.3389/fpls.2021.633979, PMID: 33692818 PMC7937975

[B29] Mizuno T. Yabuya T. Kitajima J. Iwashina T. (2013). Identification of novel C-glycosylflavones and their contribution to flower colour of the Dutch iris cultivars. Plant Physiol. Biochem. 72, 116–124. doi: 10.1016/j.plaphy.2013.06.028, PMID: 23891439

[B30] Nakatsuka T. Saito M. Yamada E. Fujita K. Kakizaki Y. Nishihara M. (2012). Isolation and characterization of *GtMYBP3* and *GtMYBP4*, orthologues of R2R3-MYB transcription factors that regulate early flavonoid biosynthesis, in gentian flowers. J. Exp. Bot. 63, 6505–6517. doi: 10.1093/jxb/ers306, PMID: 23125348 PMC3504500

[B31] Nakatsuka T. Sasaki N. Nishihara M. (2014). Transcriptional regulators of flavonoid biosynthesis and their application to flower color modification in Japanese *gentians*. Plant Biotechnol. 31, 389–399. doi: 10.5511/plantbiotechnology.14.0731a

[B32] Ni L. Wang J. Zhou F. Chen Z. (2025). Integrated multi-omics reveals Li-miR828z-*LiMYB114* regulatory module controlling anthocyanin biosynthesis during flower color development in *Lagerstroemia indica*. Ind. Crops Prod. 234, 121524. doi: 10.1016/j.indcrop.2025.121524

[B33] Nie C. Wang Y. Chen F. Fang X. Wang F. Mao M. . (2025). Spatial-temporal distribution pattern of flower color in flowering plants of Gongga Mountain and their influencing factors. Acta Agric. Universitatis Jiangxiensis 47, 373–384. doi: 10.3724/aauj.2025033

[B34] Ou Z. Luo J. Qu Y. (2024). Exploring the molecular mechanism of coloration differences in two *Meconopsis wilsonii* subspecies: *australis* and *orientalis*. Dev. Biol. 505, 1–10. doi: 10.1016/j.ydbio.2023.10.003, PMID: 37838025

[B35] Peng J. Xue C. Dong X. Zeng C. Wu Y. Cao F. (2021). Gene cloning and analysis of the pattern of expression of the transcription factor *HymMYB2* related to blue flower formation in *Hydrangea macrophylla*. Euphytica 217, 115. doi: 10.1007/s10681-021-02839-3

[B36] Qu Y. Ou Z. Wang S. (2022). Coloration differences in three *Meconopsis* species: *M. punicea*, *M. integrifolia* and *M. wilsonii*. South Afr. J. Bot. 150, 171–177. doi: 10.1016/j.sajb.2022.07.016

[B37] Qu Y. Ou Z. Yang F.-S. Wang S. Peng J. (2019). The study of transcriptome sequencing for flower coloration in different anthesis stages of alpine ornamental herb (*Meconopsis* ‘Lingholm’). Gene 689, 220–226. doi: 10.1016/j.gene.2018.12.017, PMID: 30572099

[B38] Quattrocchio F. Verweij W. Kroon A. Spelt C. Mol J. Koes R. (2006). *PH4* of *Petunia* is an R2R3 MYB protein that activates vacuolar acidification through interactions with basic-helix-loop-helix transcription factors of the anthocyanin pathway. Plant Cell 18, 1274–1291. doi: 10.1105/tpc.105.034041, PMID: 16603655 PMC1456866

[B39] Sagheer A. Chen J. Chen G. Huang J. Zhou Y. Zhao K. . (2022). Why black flowers? An extreme environment and molecular perspective of black color accumulation in the ornamental and food crops. Front. Plant Sci. 13. doi: 10.3389/fpls.2022.885176, PMID: 35498642 PMC9047182

[B40] Tan J. Wang M. Tu L. Nie Y. Lin Y. Zhang X. (2018). The flavonoid pathway regulates the petal colors of cotton flower. PloS One 8, e72364. doi: 10.1371/journal.pone.0072364, PMID: 23951318 PMC3741151

[B41] Tanaka M. Fujimori T. Uchida I. Yamaguchi S. Takeda K. (2001). A malonylated anthocyanin and flavonols in blue *Meconopsis* flowers. Phytochemistry 56, 373–376. doi: 10.1016/S0031-9422(00)00357-5, PMID: 11249104

[B42] Tanaka Y. Tsuda S. Kusumi T. (1998). Metabolic engineering to modify flower color. Plant Cell Physiol. 39, 1119–1126. doi: 10.1093/oxfordjournals.pcp.a029312

[B43] Tatsuzawa F. Tanikawa N. Nakayama M. (2017). Red-purple flower color and delphinidin-type pigments in the flowers of *Pueraria lobata* (Leguminosae). Phytochemistry 137, 52–56. doi: 10.1016/j.phytochem.2017.02.004, PMID: 28189342

[B44] Tian Z. Qiao Q. Zhang R. Wang J. Zeng Z. Wu X. . (2025). Genomic analysis of *Dasiphora* on the Qinghai-Tibet Plateau provides insights into genetic divergence and flower color variation. Plant J. 124, e70530. doi: 10.1111/tpj.70530, PMID: 41169030

[B45] Wang H. Chen X. Yong Q. Zhou L. Qu Y. (2024). Effect of petal structure and pigment composition on color formation of three species of *meconopsis*. J. Nucl. Agric. Sci. 38, 1057–1064. doi: 10.11869/j.issn.1000-8551.2024.06.1057

[B46] Wang H. Li T. Luo J. Qu Y. (2023). Cloning and expression analysis of *FLS* gene in three different color *Meconopsis* species. Plant Physiol. J. 59, 2063–2073. doi: 10.13592/j.cnki.ppj.100653

[B47] Wang Y. Zhou L. Wang Y. Liu S. Geng Z. Song A. . (2021). Functional identification of a flavone synthase and a flavonol synthase genes affecting flower color formation in *Chrysanthemum morifolium*. Plant Physiol. Biochem. 166, 1109–1120. doi: 10.1016/j.plaphy.2021.07.019, PMID: 34328869

[B48] Weiss D. (2000). Regulation of flower pigmentation and growth: Multiple signaling pathways control anthocyanin synthesis in expanding petals. Physiol. Plant. 110, 152–157. doi: 10.1034/j.1399-3054.2000.110202.x

[B49] Winkel Shirley B. (2001). Flavonoid biosynthesis. A colorful model for genetics, biochemistry, cell biology, and biotechnology. Plant Physiol. 126, 485–493. doi: 10.1104/pp.126.2.485, PMID: 11402179 PMC1540115

[B50] Xiao W. Simpson B. B. (2017). A new infrageneric classification of *meconopsis* (Papaveraceae) based on a well-supported molecular phylogeny. Syst. Bot. 42, 226–233. doi: 10.1600/036364417X695466

[B51] Xue Y. Zhao Q. Huang Y. Liu Z. Wu L. Xi C. . (2015). Research on relationship between floral colors and intracellular environment of *camellia reticulata* lindl. J. Yunnan Agric. Univ. China 30, 455–463. doi: 10.16211/j.issn.1004-390X(n).2015.03.022

[B52] Yang K. Hou Y. Wu M. Pan Q. Xie Y. Zhang Y. . (2023). *DoMYB5* and *DobHLH24*, transcription factors involved in regulating anthocyanin accumulation in *Dendrobium officinale*. Int. J. Mol. Sci. 24, 7552. doi: 10.3390/ijms24087552, PMID: 37108715 PMC10142772

[B53] Yang Y. Li B. Feng C. Wu Q. Wang Q. Li S. . (2020). Chemical mechanism of flower color microvariation in *Paeonia* with yellow flowers. Hortic. Plant J. 6, 179–190. doi: 10.1016/j.hpj.2020.04.002

[B54] Ye S. Hua S. Ma T. Ma X. Chen Y. Wu L. . (2022). Genetic and multi-omics analysis reveal *bnaA07.PAP2^In-184–317^* as the key gene conferring anthocyanin-based color in *brassica napus* flowers. J. Exp. Bot. 73, 6630–6645. doi: 10.1093/jxb/erac312, PMID: 35857343

[B55] Yin X. Wang T. Zhang M. Zhang Y. Muhammad I. Chen L. . (2021). Role of core structural genes for flavonoid biosynthesis and transcriptional factors in flower color of plants. Biotechnol. Biotechnol. Equip. 35, 1214–1229. doi: 10.1080/13102818.2021.1960605

[B56] Yoshida K. Kitahara S. Ito D. Kondo T. (2006). Ferric ions involved in the flower color development of the Himalayan blue poppy, *Meconopsis grandis*. Phytochemistry 67, 992–998. doi: 10.1016/j.phytochem.2006.03.013, PMID: 16678868

[B57] Yoshida K. Mori M. Kondo T. (2010). Blue flower color development by anthocyanins: from chemical structure to cell physiology. Natural Prod. Rep. 26, 884–915. doi: 10.1039/b800165k, PMID: 19554240

[B58] Yu P. Piao M. Kong X. Liu Y. Gao L. Huang Y. . (2024). The coordinated interaction or regulation between anthocyanin and carotenoid pathways in OT hybrid lilies based on metabolome and time-course transcriptomics analysis. Ind. Crops Prod. 222, 119795. doi: 10.1016/j.indcrop.2024.119795

[B59] Yu W. Yang Z. Li J. Li X. Wang G. Xing Z. (2020). Garden applications and research progress of *meconopsis* plants. J. Sichuan Forestry Sci. Technol. 41, 115–121. doi: 10.12172/201910310003

[B60] Yuan M. Ma Y. Wu R. Kang X. Ding C. Du L. (2024). Physicochemical characteristics and anthocyanin components affecting the color of rose petal. Acta Botanica Boreali-Occi Dentalia Sin. 44, 255–269. doi: 10.7606/j.issn.1000-4025.20230582

[B61] Zan W. Wu Q. Dou S. Wang Y. Zhu Z. Xing S. . (2024). Analysis of flower color diversity revealed the co-regulation of cyanidin and peonidin in the red petals coloration of *Rosa rugosa*. Plant Physiol. Biochem. 216, 109126. doi: 10.1016/j.plaphy.2024.109126, PMID: 39288572

[B62] Zhai Y. Lv J. Li X. Luo X. Li L. Shi Q. (2020). Effects of Cell Sap pH on the Flower Color Formation in *Primula vulgaris*. Acta Hortic. Sin. 47, 477–491. doi: 10.16420/j.issn.0513-353x.2019-0228

[B63] Zhao D. Tao J. (2015). Recent advances on the development and regulation of flower color in ornamental plants. Front. Plant Sci. 6. doi: 10.3389/fpls.2015.00261, PMID: 25964787 PMC4410614

[B64] Zhao D. Tao J. Han C. Ge J. (2012). Flower color diversity revealed by differential expression of flavonoid biosynthetic genes and flavonoid accumulation in herbaceous peony (*Paeonia lactiflora* Pall.). Mol. Biol. Rep. 39, 11263–11275. doi: 10.1007/s11033-012-2036-7, PMID: 23054003

[B65] Zhou X. Fan Z. Chen Y. Zhu Y. Li J. Yin H. (2013). Functional analyses of a flavonol synthase-like gene from *Camellia nitidissima* reveal its roles in flavonoid metabolism during floral pigmentation. J. Biosci. 38, 593–604. doi: 10.1007/s12038-013-9339-2, PMID: 23938391

[B66] Zhou N. Yan Y. Wen Y. Zhang M. Huang Y. (2023). Integrated transcriptome and metabolome analysis unveils the mechanism of color-transition in E*dgeworthia chrysantha* tepals. BMC Plant Biol. 23, 567. doi: 10.1186/s12870-023-04585-1, PMID: 37968605 PMC10652483

[B67] Zou J. Zeng X. Chen H. Cai X. Wang C. (2017). Analysis on characteristic color compounds in different varieties of *Osmanthus fragrans* Lour. during flowering and senescence. J. South. Agric. 48, 1683–1690. doi: 10.3969/j.issn.2095-1191.2017.09.24

