# Supplementary figures and images for: Integrated metabolomic and transcriptomic analyses reveal distinct flavonoid biosynthetic pathways underlying petal color diversity in Meconopsis
Source: Front Plant Sci. 2026 Feb 17;17:1743820. doi: 10.3389/fpls.2026.1743820 (PMC12955549; doi:10.3389/fpls.2026.1743820)

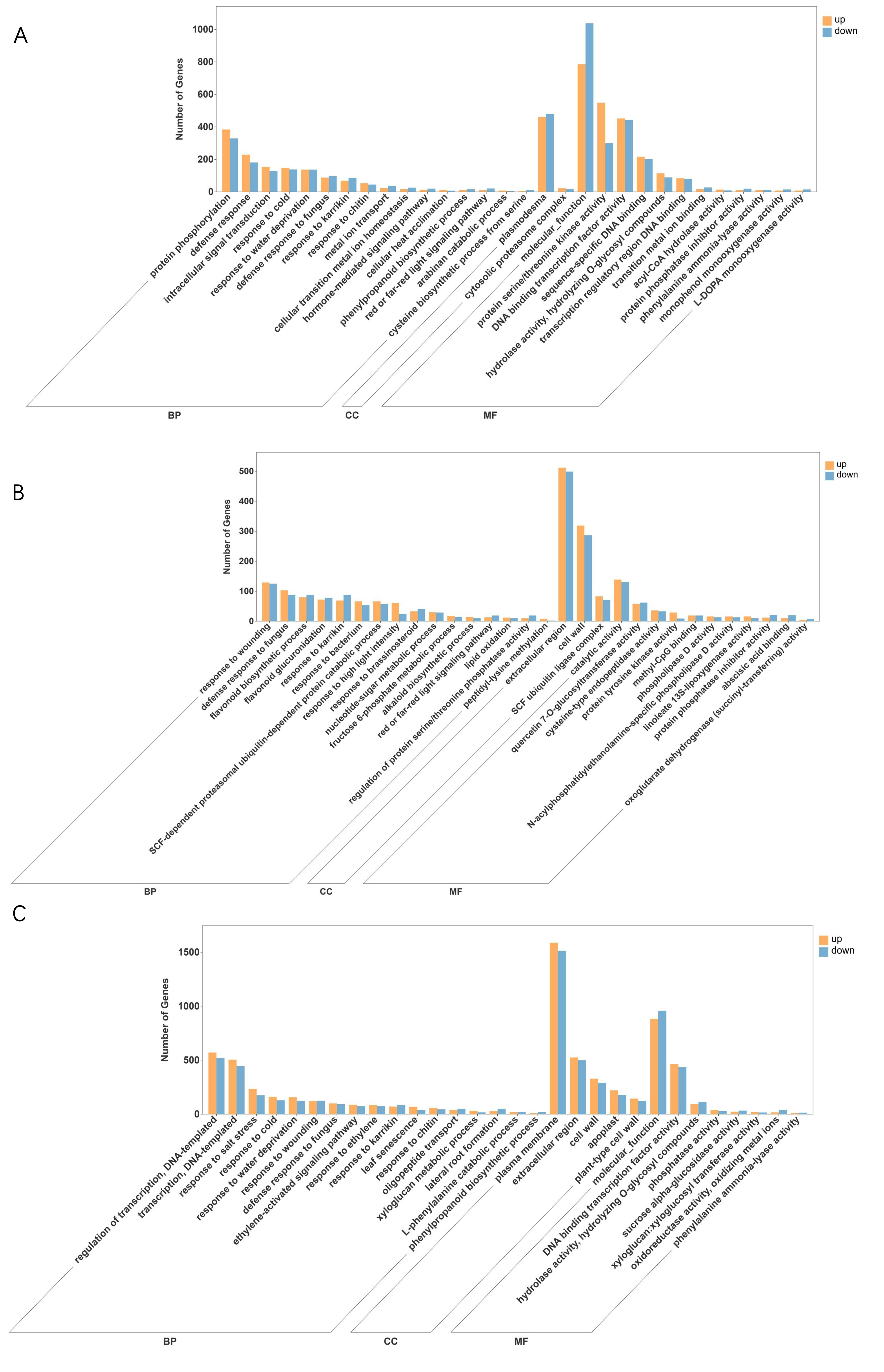

Supplement: Supplementary Figure 3 — GO enrichment analysis of DEGs. [file Image3.tif]

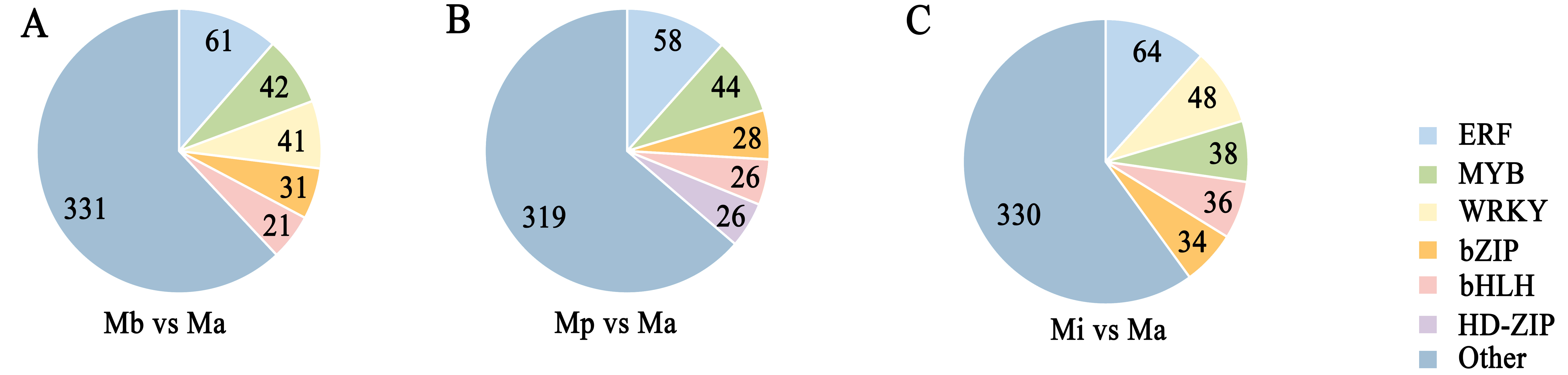

Supplement: Supplementary Figure 4 — Differential Transcription Factor Classification Chart. [file Image4.tif]
